# Coagulopathy of Dengue and COVID-19: Clinical Considerations

**DOI:** 10.3390/tropicalmed7090210

**Published:** 2022-08-25

**Authors:** Amin Islam, Christopher Cockcroft, Shereen Elshazly, Javeed Ahmed, Kevin Joyce, Huque Mahfuz, Tasbirul Islam, Harunor Rashid, Ismail Laher

**Affiliations:** 1 Department of Haematology, Mid & South Essex University Hospital NHS Foundation Trust, Prittlewell Chase, Westcliff-on-Sea SS0 0RY, UK; 2 Department of Haematology, Queen Mary University of London, Mile End Road, London E1 3NS, UK; 3 Adult Haemato-Oncology Unit, Faculty of Medicine, Ainshams University, Cairo 11566, Egypt; 4 Department of Microbiology and Virology, Mid & South Essex University Hospital NHS Foundation Trust, Westcliff-on-Sea SS0 0RY, UK; 5 Department of Haematology and Oncology, Combined Military Hospital, Dhaka 1206, Bangladesh; 6 Department of Pulmonology and Critical Care Medicine, Indiana School of Medicine, Lafayette, IN 47907, USA; 7 National Centre for Immunisation Research and Surveillance, The Children’s Hospital at Westmead, Westmead, NSW 2145, Australia; 8 Sydney Institute for Infectious Diseases, The University of Sydney, Westmead, NSW 2145, Australia; 9 Department of Anesthesiology, Pharmacology & Therapeutics, Faculty of Medicine, The University of British Colombia, Vancouver, BC V6T 1Z3, Canada

**Keywords:** COVID-19, cross-reactivity, dengue, haemorrhage, thrombocytopenia, thrombosis

## Abstract

Thrombocytopenia and platelet dysfunction commonly occur in both dengue and COVID-19 and are related to clinical outcomes. Coagulation and fibrinolytic pathways are activated during an acute dengue infection, and endothelial dysfunction is observed in severe dengue. On the other hand, COVID-19 is characterised by a high prevalence of thrombotic complications, where bleeding is rare and occurs only in advanced stages of critical illness; here thrombin is the central mediator that activates endothelial cells, and elicits a pro-inflammatory reaction followed by platelet aggregation. Serological cross-reactivity may occur between COVID-19 and dengue infection. An important management aspect of COVID-19-induced immunothrombosis associated with thrombocytopenia is anticoagulation with or without aspirin. In contrast, the use of aspirin, nonsteroidal anti-inflammatory drugs and anticoagulants is contraindicated in dengue. Mild to moderate dengue infections are treated with supportive therapy and paracetamol for fever. Severe infection such as dengue haemorrhagic fever and dengue shock syndrome often require escalation to higher levels of support in a critical care facility. The role of therapeutic platelet transfusion is equivocal and should not be routinely used in patients with dengue with thrombocytopaenia and mild bleeding. The use of prophylactic platelet transfusion in dengue fever has strained financial and healthcare systems in endemic areas, together with risks of transfusion-transmitted infections in low- and middle-income countries. There is a clear research gap in the management of dengue with significant bleeding.

## 1. Introduction

The exponential spread of COVID-19 that started early in 2020 resulted in one of the worst global pandemics of our lifetime, leading to a gauntlet of clinical challenges, including responding to unique patterns of coagulation anomalies. The COVID-19 outbreak has further added to clinical challenges in tropical and subtropical regions of the world, where dengue fever, caused by dengue virus (DENV) is also endemic. Similarities in clinical manifestations of COVID-19 and dengue fever often create diagnostic dilemmas in dengue-endemic countries with limited resources, often leading to delayed or even incorrect diagnosis. Furthermore, there are growing concerns of cross-reactivity due to pre-existing DENV-antibodies that potentially can enhance COVID-19 antibody-dependent immune responses [1]. Mild to moderate thrombocytopenia are common to both conditions but have different clinico-pathological aetiologies and treatment approaches, making earlier diagnosis key to preventing severe outcomes. Moreover, despite mild to moderate thrombocytopenia in COVID-19 diseases, anticoagulation is an integral part of the treatment protocols of COVID-19 [2]. In contrast, treatment with anticoagulants is contraindicated in dengue infections and non-steroidal anti-inflammatory agents are also avoided merely because of their theoretic risk of aggravated bleeding such as from the gastrointestinal tract [3]. There are concerns about the indiscriminate use of prophylactic platelet transfusions in dengue viral infections, especially in tropical dengue-endemic areas where the availability of safe transfusion services remains a significant challenge due to greater risks of transfusion-transmitted infections and other immunological concerns.

DENVs are the most important human arboviruses worldwide. Transmission of dengue occurs via Aedes mosquitoes, producing four antigenically distinct serotypes (DENV-1 to DENV-4). Clinical presentations range from mild to more severe infections, with significant morbidities and mortalities in endemic areas. It is estimated that there are currently 50–100 million cases of dengue infections worldwide annually, with more than 500,000 reported cases of severe forms of dengue infections such as dengue haemorrhagic fever (DHF) and dengue shock syndrome (DSS) [4]. The immunological cross-reactivity and common pathological processes, such as capillary leakage, thrombocytopenia, and coagulopathy, between SARS-CoV-2 and DENV, make it difficult to distinguish their shared clinical and laboratory characteristics (Table 1) [1]. Patients with dengue fever, including those with positive non-structural protein 1 (NS1) and/or IgM serology results, should be differentiated from those with SARS-CoV-2 infection, and if necessary, dengue IgM/IgG testing should be repeated to identify co-infection or serological overlap [5]. It is necessary to prepare for dengue outbreaks alongside controlling the COVID-19 pandemic, as a resurgence poses a very real threat [6].

## 2. Virology of DENV

DENV is a single-stranded RNA virus of the Flaviviridae family. Each of the four DENV serotypes vary in their epidemiological patterns, but some countries may have hyperendemicity with more than one serotype actively circulating at the same time [4], as in the Indian subcontinent and South East Asia. There may be different genotypes with minor antigenic changes within the same serotype. Life-long immunity develops after infection with the same serotype, but with only a partial and short-lived immunity to another serotype. The recorded number of confirmed dengue cases may not accurately reflect the true burden of dengue, as many patients are asymptomatic or remain undiagnosed.

The gold standard test to confirm dengue is by reverse transcriptase-PCR (rt-PCR) targeting regions within the genome of DENV. Resource-limited countries rely on detecting DENV IgG and IgM. The serology tests could cross-react with other flaviviruses; hence the mainstay is testing for active viremia by rt-PCR [9]. Antigenic assays have recently been developed that target NS1 which is secreted in infected mammalian cells. A serotype-specific mAb-based NS1 antigen-capture ELISA has reliable serotype specificity [10]. Whilst the cross-reactivity is observed in serological assays of DENV, there is no evidence that an infection with DENV confers cross-infectivity to other viruses such as West Nile virus, Japanese Encephalitis virus, or other arthropod-borne flaviviruses. An exception is cross-reactivity of DENV with yellow fever virus, the underlying cause of which remains unknown.

## 3. Virology of COVID-19

COVID-19 is caused by SARS-CoV-2, of the order Nidovirales within the family Coronaviridae. It is an enveloped, positive-sense single-stranded RNA (+ssRNA) virus of dimension between 65 and 125 nm [8]. Cell membrane attachment is achieved through a spike protein interaction with cell surface receptor angiotensin-converting enzyme receptor 2 (ACE2), leading to membrane fusion and deposition of the virion’s genetic material into the cytoplasm.

New sub-variants of SARS-CoV-2 have emerged with varying clinical significance, including transmission rates and susceptibility to vaccination [11,12]. As a whole, SARS-CoV-2 shows structural similarities to two of its relatives, SARS-CoV (Severe Acute Respiratory Syndrome-Coronavirus) and MERS-CoV (Middle Eastern Respiratory Syndrome-Coronavirus) [11] both of which have caused recent epidemics.

## 4. Epidemiology of Dengue

Dengue is the second most diagnosed cause of fever after malaria in travelers returning from low- and middle-income countries. The global incidence of dengue continues to grow, even though most cases are asymptomatic or mild and self-managed, suggesting that case numbers are likely under-reported. Many cases are also misdiagnosed as other febrile illnesses [13]. Dengue is endemic in 129 countries with 390 million cases globally per year, of which 96 million manifest clinically, with the majority (70%) of the burden being in Asia [14,15]. Dengue is now spreading to new areas including Europe, with explosive outbreaks also occurring. There were 2000 cases of dengue in the Madeira Islands of Portugal in 2012, with imported cases detected in mainland Portugal and 10 other European countries.

The largest recent outbreak of dengue was in 2019, when all the World Health Organization (WHO) Regions were affected. The Region of the Americas alone reported 3.1 million cases, with more than 25,000 cases classified as severe [16]. Dengue affected several countries in 2020; however, cases drastically reduced by 55–65% with the advent of the COVID-19 wave in the year 2021 across the globe indicating an ‘inverse relationship’ between the two diseases [17].

Lifestyle changes and globalisation place severe limitations on the control of mosquito vectors to reduce dengue viral infections, in part due to rapid urbanisation in many counties. The increasing use of containers for water storage, such as automobile tyres and plastic containers, creates ideal sites for oviposition and larval habitats for *Aedes aegypti* mosquitoes. Most of the mosquito-control efforts directed at adult mosquitoes in the early 1970s used expensive methods that were largely ineffective. Changing human lifestyles creates more larval habitats, thus facilitating dengue transmission by increasing mosquito populations in areas with crowded human habitation [18].

## 5. Epidemiology of COVID-19

COVID-19 was first described in Wuhan province (China) in December 2019. It quickly spread throughout the world and was designated a Public Health Emergency of International Concern (PHEIC), the WHO’s highest level of alert, on 30 January 2020. The resultant pandemic created dramatic lockdown measures across the globe that affected international and local travel. There were over 541 million confirmed COVID-19 cases and 6.3 million deaths reported to the WHO as of 4 July 2022 [19].

SARS-CoV-2 is believed to have crossed the species barrier to humans from an animal reservoir, most likely from bats [20]. Transmission is believed to occur either through large droplets or aerosols in the form of coughing or sneezing [21]. As such, the infection spreads best in densely packed communities, especially where physical distancing is not practiced, and where cough etiquette is not observed meticulously. There are also rare reports of faeco-oral or vertical transmissions [22].

The incubation period is variably reported to be between 2 and 14 days [22,23]. The period of infectivity extends from a few days before the symptom onset to several days post-symptom resolution [24]. Such a lag before the onset of warning signs and the cessation of infectivity, along with cases of asymptomatic spreading [25], allows COVID-19 to continue to spread even in a population vigilant of the signs and practicing symptomatic isolation.

The spectrum of COVID-19 severity varies from asymptomatic to life-threatening/fatal infection. Risk factors for severe infection include advanced age and presence of comorbidities [22] (Table 2). Vaccinations have become the leading deterrent for severe illness and hospitalisation [26].

At the time of writing, the global outbreak of COVID-19 is largely receding, with the last peak recorded in January 2022 [19]. The WHO regions with the greatest burden of disease are the Americas, Europe and the Western Pacific region; however, inconsistencies in global surveillance and reporting systems are likely to misrepresent the true magnitude of the pandemic.

## 6. Severe Dengue in Adults and Infants

The risk of dengue infection in various patient groups is summarised in Table 2.

### 6.1. Antibody-Dependent Enhancement and Severe Dengue

Severe dengue most commonly occurs in infants and adults with secondary dengue infections (i.e., infection with a DENV type different from a previous DENV infection). The most widely cited hypothesis for this is antibody-dependent enhancement (ADE) of disease, which occurs when non-neutralising anti-DENV antibodies bind to, but do not neutralise, an infecting DENV. This virus-antibody complex allows for enhanced viral entry into host cells (specifically dendritic cells and macrophages) and the virus replicates and generates higher viral titers in blood than when the anti-DENV antibody is not present, resulting in a ‘cytokine storm’ and exacerbating the disease.

### 6.2. Severe Dengue among Infants

Infants in dengue-endemic areas have anti-DENV IgG antibodies at birth. Anti-DENV IgG antibodies are passed from a mother to foetus (IgM does not cross placenta). This passively transferred maternal anti-DENV IgG can protect the infant for a few months after birth, which can explain why the occurrence of dengue in infants under 4 months of age is unusual. However, as the maternal anti-DENV IgG titer falls 4–6 months after birth, ADE outweighs neutralisation, and the infant is at higher risk for severe disease even with a primary DENV infection. Children aged one year or more are not at increased risk.

## 7. General Aspects of Platelets and Haemostasis

Platelets play an integral part in primary haemostasis by forming a thrombus at the site of vascular injury; new platelets are produced daily to maintain a platelet count of 150–400 × 10^9^ platelets/L blood [30,31,32]. Activated platelets undergo actin-mediated shape changes (from smooth discoid to spiny spheres) when passing through damaged blood vessels. Various receptors for adhesive and clotting proteins in activated platelets then attract other platelets to form a plug that limits vascular leakage [33].

Platelets activate neutrophils, monocytes and lymphocytes to form platelet-leukocyte aggregates, and thus participate in immune responses. It is possible that platelet surface receptors such as toll-like receptors (TLRs) and glycoprotein V1 play roles in immune responses [34]. A haemostatic envelope which prevents excessive bleeding after vascular injury is formed by exposure to membrane-bound tissue factor (TF) that is constitutively expressed on the cell surfaces of fibroblasts and muscle cells [35]. The conversion of factor X to Xa occurs after catalysis by TF-V11a, which further assembles the prothrombinase complex formed by factor Xa, factor Va, factor II (prothrombin) and Ca^2+^, resulting in the generation of thrombin [36]. The semipermeable properties of endothelial cells are supported by platelets in a well-recognised process, where platelet activation releases proangiogenic molecules that mediate the migration and proliferation of vascular cells, and vessel organisation and stabilisation [37,38].

## 8. Coagulopathy in Dengue

Thrombocytopenia remains a potential indicator of clinical severity of dengue infection as per WHO guidelines, with the most recent WHO guidelines (from 2009) describing rapid decreases in platelet count, or a count of less than 150,000 per microliter of blood [39]. Coagulation and fibrinolytic pathways are activated during an acute dengue infection [40]. Thrombocytopenia, coagulopathy and vasculopathy are related to the platelet and endothelial dysfunction observed in severe dengue. A recent study reports that mild to moderate thrombocytopenia occurs 3 to 7 days (significantly on the 4th day) after infection and returns to normal levels on day 8 or 9 of infections in adult patients without shock [41]. There is no clear relationship between the platelet count, disease severity and bleeding manifestations in dengue viral infections in children [42]. A platelet count of 5 × 10^9^/L and haematocrit (HCT) > 50 L/L (normal range 0.40–0.52) is associated with bleeding symptoms in adults, though a study of 245 dengue patients showed there is no clear correlation between clinical bleeding and platelet count, whereas 81 non-bleeding patients had a platelet count of <20 × 10^9^/L [43], while another study of 225 patients demonstrated bleeding to be more frequent when the platelet count reached below 20 × 10^9^/L [44].

Most clinical guidelines recommend that platelet transfusions should be given to patients who develop serious haemorrhagic symptoms or when platelet count is below 10–20 × 10^9^/L without haemorrhage. It is also advised that platelet transfusion should be considered in patients with bleeding manifestations when platelet count is below 50 × 10^9^/L. However, the efficacy of platelet transfusions in dengue viral infection remains a matter of debate. A study involving 106 children with DSS who had thrombocytopenia and coagulopathy indicated no significant differences in bleeding manifestations between children who received platelets or not. Patients who received platelet transfusions had more transfusion-associated pulmonary oedema and longer hospital stays [45].

Severe thrombocytopenia and the secondary effects of hypoxia due to prolonged shock resulting in acidosis can trigger disseminated intravascular coagulation (DIC) and major haemorrhagic manifestations in some patients. Despite less severe bleeding and some minor abnormalities of basic clotting tests, all major pathways of the coagulation cascade are changed in children with DSS. Levels of proteins C, S and antithrombin are reduced secondary to leakage through the vascular endothelium, and correlate with the severity of shock in critical dengue infections. Direct activation of fibrinolysis by DENV may be secondary to raised levels of TF, thrombomodulin and plasminogen activator inhibitor 1 (PAI-1) which can then activate endothelial cells, platelets and monocytes.

The details of the pathogenesis of thrombocytopenia and bleeding are poorly understood, with many hypotheses offered on the pathogenesis, such as that DENV directly or indirectly affects bone marrow progenitor cells by inhibiting their function and reducing proliferative capacities [46], as supported by findings that DENV induces bone marrow hypoplasia during acute phases of the disease [47]. In addition to low platelet counts, functional disruption of platelets is associated with deregulation of the plasma kinin system and the immunopathogenesis of dengue [48]. Dengue viral infection induces platelet consumption due to DIC, platelet destruction due to increased apoptosis, lysis by the complement system and activation of antiplatelet antibodies [49]. Cytokines (e.g., TNF-α), interleukins (IL-2, IL-6, IL-8) and interferons (IFN-α and IFN-γ) also have roles in thrombocytopenia by suppressing haematopoiesis. Levels of these cytokines correlate with the clinical severity of dengue infection [50].

Other comorbidities increase the risk of severity of dengue; for example, allergy or diabetes increase the risk of DHF by 2.5 times, and hepatitis also increases the risk of complications. Increases in viral infections, hyperferritinaemia and the activation of coagulation and fibrinolytic systems occur in children with dengue compared to those without hyperferritinaemia. Other contributing factors to thrombocytopenia include cytokines, coagulation mediators, adhesive molecules and proteins, which encourage inflammatory response promoting cell interactions between platelets, immune cells and the endothelium. In addition, thrombocytopenia resulting from decreased bone marrow production and increased peripheral destruction of platelets, causes immune thrombocytopenia (ITP).

NS1 correlates well with levels of viremia, and is particularly high in patients suffering from DHF. Several mechanisms have been proposed by which NS1 contributes to the coagulopathy seen in DHF, such as proinflammatory cytokine release via activation of macrophages, and activation of complement, whilst expressed on the surface of infected cells and when released into the surrounding plasma, both of which contribute to endothelial damage and increase permeability, leading to DHF [51]. Anti-NS1 (a cross-reactive anti-dengue antibody) and prM (structural precursor-membrane protein), and coronavirus E proteins all target platelets, endothelial cells and coagulation molecules. This process contributes to endothelial damage, macrophage activation and more platelet dysfunction, ultimately leading to further worsening of coagulopathy. Increased vascular fragility and impaired platelet function leads to haemorrhage, which can further contribute to plasma leakage in DHF/DSS [52]. There may also be other more complex mechanisms involved in dengue immunopathogenesis, platelet dysfunction and thrombocytopenia [53].

## 9. Thrombocytopenia Associated with COVID Vaccines

Thrombotic thrombocytopenia syndrome (TTS), a complication of COVID-19 vaccines, involves thrombosis and thrombocytopenia with infrequent arterial thrombosis. TTS appears to mostly affect females aged between 20 and 50 years old, with no predisposing risk factors conclusively identified so far. Cases are characterised by thrombocytopenia, higher levels of D-dimers than commonly observed in venous thromboembolic events (VTE), inexplicably low fibrinogen levels and worsening thrombosis. Hyper-fibrinolysis associated with bleeding can also occur. Antibodies that bind platelet factor 4, like those associated with heparin-induced thrombocytopenia, have also been identified but in the absence of patient exposure to heparin treatment. TTS is an extremely rare but increasingly recognised serious adverse event related to thromboembolism at unusual sites, such as cerebral venous sinus thrombosis (CVST) or abdominal thromboses (splanchnic, mesenteric or portal vein), all of which are associated with thrombocytopenia. ‘CVST with thrombocytopenia’ is a rare subtype of cerebrovascular accident, with an incidence of 5.0 per million in those receiving Vaxzevria (manufactured by AstraZeneca) and 4.1 per million in those receiving mRNA-based vaccines, and the prevalence is three times greater in younger to middle aged women (mean age 35). Several countries have suspended the use of adenovirus-vectored vaccines for younger individuals. The prevailing opinion is that the risk of developing COVID-19 disease, including thrombosis, far exceeds the extremely low risk of TTS associated with highly efficacious vaccines. Mass vaccination should continue but with caution. Vaccines that are more likely to cause TTS (e.g., Vaxzevria) should be avoided in younger patients for whom an alternative vaccine is available [54]. The only vaccine currently known to cause ITP is the mumps, measles and rubella (MMR) vaccine, but with low incidence.

## 10. Coagulopathy of Dengue versus COVID-19

The clinical manifestation and magnitude of coagulopathy varies greatly in patients with dengue due to differences in viral virulence, routes of exposure and host conditions [55]. Minor bleeding, including petechiae, epistaxis and bleeding gums, can occur and help to recognise viral haemorrhagic fever in its early stages [56]. Unlike COVID-19, dengue rarely causes respiratory dysfunction and/or acute lung injury. Vascular injury results in increased permeability, hypovolaemia and circulatory shock in the advanced stages of severe viral hemorrhagic fever.

Shock can also occur in COVID-19. Multisystem inflammatory syndrome in children (MIS-C) and multisystem inflammatory syndrome in adults (MIS-A) are rare post-infectious complications characterised by fever, systemic inflammation, abdominal pain and cardiac involvement. The symptoms usually occur late, while the sudden onset of severe systemic inflammation with shock is reminiscent of toxic shock syndrome in bacterial infections. The aetiology of MIS-C and MIS-A is uncertain, but derangement of the autoimmune reaction is a possibility [57]. Increased vascular permeability in viral hemorrhagic fever also induces coagulation defects that can result in severe bleeding [58].

Systemic viral infection also induces an acute inflammatory and hypercoagulable state, causing DIC that increases the risk of multiorgan failure and death. However, except for Ebola and Marburg, bleeding in haemorrhagic fevers is rarely a direct cause of death [59]. Coagulopathy is common in filovirus diseases and also occurs in dengue. Petechiae, gingival and mucosal bleeding, and sustained bleeding at venipuncture site can occur in these patients. These symptoms usually diminish within a week, but a small proportion of patients develop DHF with worsening of bleeding and shock [60]. In 1997, the WHO characterised typical DHF by four major clinical manifestations: (1) sustained high fever for two to seven days; (2) a haemorrhagic tendency, such as a positive tourniquet test, or clinical bleeding; (3) thrombocytopenia (platelets ≤ 100 × 10^9^/L); and (4) evidence of plasma leakage manifested by haemoconcentration (>20% increase in hematocrit) or pleural effusion [61].

The clinical features of coagulation disorders are quite different in COVID-19 and dengue (see Table 3). COVID-19 is characterised by a high prevalence of thrombotic complications, with an estimated overall prevalence of VTE of 14.1% [62]. The incidence of VTE in COVID-19 is at least threefold higher than in other viral respiratory infections [63]. In more critically ill patients, the incidence of VTE is 45.6%, while it was 23.0% in non-ICU (intensive care unit) patients [62]. Coagulopathy in COVID-19 is initiated by local lung injury, and following the initial localised thrombo-inflammatory response, systemic hypercoagulability becomes prominent. Coagulation tests including prothrombin time (PT) and activated partial thromboplastin time (aPTT) are usually normal; however, more sensitive viscoelastic testing demonstrates a hypercoagulable pattern mainly due to activated platelets [64]. Since SARS-CoV-2 injures vascular endothelial cells, the loss of anticoagulant activity is another critical factor for prothrombotic changes. Internalisation of ACE2 to increase angiotensin II levels causes vasoconstriction, hyperinflammation, and the release of prothrombotic substances such as von Willebrand factor (VWF), P-selectin, factor VIII and angiopoietin 2 [65]. Bleeding rarely occurs in COVID-19, especially in advanced stages of critical illness. Increased haemorrhage is due to thrombocytopenia, platelet dysfunction and consumptive coagulopathy often complicated by secondary infections [66].

## 11. Prior Exposure to DENV and COVID-19 Severity

It is possible that prior exposure to DENV could provide some degree of cross-protection to SARS-CoV-2 infection, rendering it less severe in regions where dengue is endemic, as supported by a report from Singapore where a man and a woman (both aged 57 years) were originally COVID-19 virus-positive and false-positive in serological tests for dengue, including DENV-IgM and IgG [68]. Reports are available which show that sero-diagnostic tests for DENV yield false-positive results for SARS-CoV-2 and vice versa in dengue-endemic regions, thereby indicating potential cross-reactivity between the viruses. A recent study that tested the antigenic similarities of SARS-CoV-2 and DENV using computational docking demonstrated that human DENV antibodies can bind to the receptor binding domain of SARS-CoV-2 spike protein. Some of these interactions can also potentially intercept human ACE2 receptor binding to the receptor-binding motif (RBM). Dengue serum samples predating the COVID-19 outbreak cross-reacted with the SARS-CoV-2 spike protein. Of importance is that the m396 and 80R antibodies (against SARS virus) did not dock with RBM of SARS-CoV-2. It is probable that immunological memory/antibodies to DENV in endemic countries could reduce the severity and spread of COVID-19. It is not known whether SARS-CoV-2 antibodies will hinder DENV infections by binding to DENV particles and reduce dengue incidence in the future, or even facilitate DENV infection by deploying ADE [69].

## 12. Clinical Manifestations

### 12.1. Haemorrhagic Manifestations of Dengue

**Figure 1:**
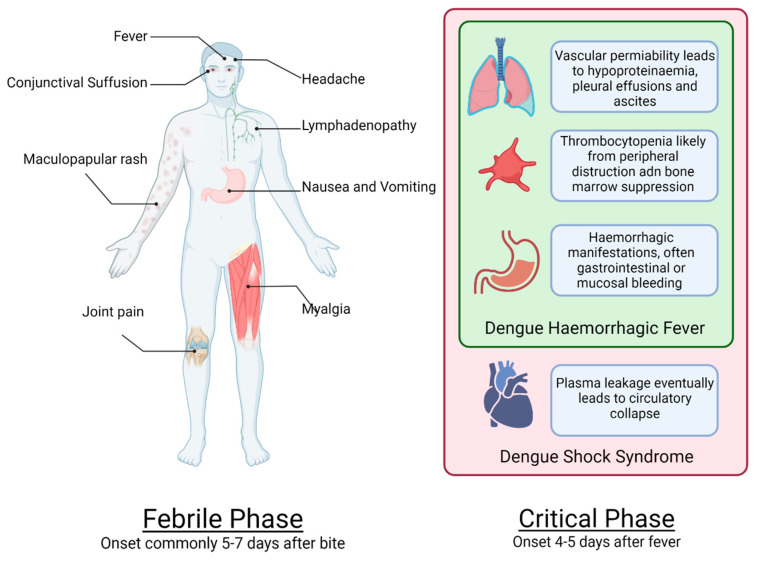
Systemic effects of dengue and time course of infection.

Minor bleeding such as that in the nose, gums and gastrointestinal tract is occasionally observed in children without shock [70]. Mucosal bleeding is more common and of greater severity in adults. However, intracranial haemorrhage is very rare but can be a fatal complication [71]. Patients with profound or prolonged shock complicated by metabolic acidosis and/or DIC typically experience gastrointestinal bleeding. The clinical features of dengue infection are summarised in Figure 1.

### 12.2. Thrombosis in COVID-19

**Figure 2:**
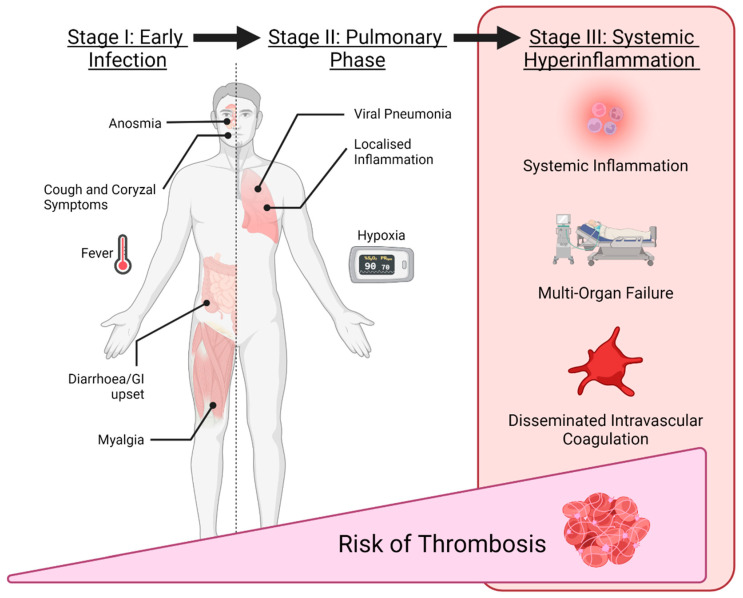
Typical stages of a COVID-19 infection and their clinical features.

Exaggerated inflammatory reactions occur during the advanced stages of COVID-19, including progression to acute respiratory distress syndrome (ARDS) and multi-organ failure, ultimately leading to shock, and the development of DIC [72]. The process is multifactorial but is believed to largely revolve around a disproportionate inflammatory cascade and cytokine storm [73] dysregulating haemostatic failsafe mechanisms. Figure 2 summarises the stages and clinical features of classical COVID-19 infections.

Several coexisting factors predisposing to increased thrombosis are observed throughout the progression of a COVID-19 infection and may manifest as thrombotic events such as myocardial infarction, pulmonary embolism or cerebrovascular disorders, with the highest rates reported among severe cases, particularly those needing ICU admission [22]. However, there are reports of thrombosis in otherwise asymptomatic individuals and occurring weeks after symptom resolution [72]. Mechanisms of coagulopathy include viral interaction with the renin angiotensin system (RAS) via its binding to ACE2 receptors in the lungs, leading to an upregulation of the prothrombotic angiotensin-II, a cytokine storm and complement activation, endothelial dysfunction and sepsis-driven and hypoxia-driven coagulopathy [72,74,75]. Severe COVID-19 has further been associated with the shutdown of normal fibrinolysis, an essential failsafe in thrombotic homeostasis [76]. This is believed to be due to increased release of antifibrinolytic agents, most notably plasminogen activator inhibitor 1 (PAI-1) from activated platelets and damaged endothelium [77]. Thus, clots not only form more easily, but are broken down less efficiently once formed. Not all these mechanisms are confined to the severely ill patients, but new mechanisms will exacerbate disease progression. Thus, the risk of thrombosis persists throughout a COVID-19 infection, with the risk of thrombosis being greater in more severely affected patients [78].

## 13. Serological Cross-Reactivity between Dengue and COVID-19

It is often difficult to distinguish between COVID-19 and dengue owing to their shared clinical and laboratory features, including possible cross-reactivity. Failing to consider COVID-19 due to false-positive dengue serology can have serious implications and vice versa. In a report published from Israel using clinical data and serum samples from 55 individuals with COVID-19, dengue-specific antibodies were detected by lateral-flow rapid tests and enzyme-linked immunosorbent assay (ELISA) in 12 (21.8%) COVID-19 patients compared to zero positive cases in a control group of 70 healthy individuals (*p* < 0.001). Of the 12 positive cases, nine had IgM positivity, two had IgG IgM positivity and one had both IgM and IgG IgM positivity. ELISA testing for dengue was positive in two additional subjects using envelope protein-directed antibodies but negative by lateral flow rapid testing. Out of 95 samples obtained from patients diagnosed with dengue before September 2019, SARS-CoV-2 serology targeting the S protein was positive/equivocal in 21 (22%) (16 IgA, 5 IgG) versus 4 positives/equivocal in 102 controls (4%) (*p* < 0.001). Subsequent in silico analysis revealed possible similarities between SARS-CoV-2 epitopes in the HR2 domain of the spike protein and the dengue envelope protein. This finding supports possible cross-reactivity between DENV and SARS-CoV-2, which can lead to false-positive dengue serology among COVID-19 patients and vice versa—which has important therapeutic and public health implications [79].

## 14. Management Recommendations for COVID-19 Coagulopathy

The best practice management of coagulopathy in the context of a COVID-19 infection continues to evolve as new evidence emerges, and no guidance can adequately cover every possible situation. However, some recommendations can be made regarding approaches to mitigate and treat coagulopathy in these patients. The scope of this paper does not cover all eventualities such as management of specific thrombotic syndromes (e.g., acute coronary syndrome) in COVID-19, but instead lays out recommendations for general prophylactic management.

### 14.1. Mild COVID-19 Infection

Anticoagulation should not be commenced routinely in patients with mild, early symptoms, as the risk of coagulation disorders is the lowest in these patients who can be treated at home [75]. Appropriate counselling should be offered, including increased risk of coagulopathy, warning signs of clotting and basic measures for prevention (e.g., staying well hydrated, staying mobile).

A risk–benefit analysis should be conducted for patients with other co-morbidities that can increase coagulation risk. Anticoagulation should be continued in those already on anticoagulant therapy (for instance, a patient with pre-existing atrial fibrillation treated with a direct-acting oral anticoagulant). However, there are specific considerations concerning the suitability of treatment in light of their current infection status, such as safe access to international normalised ratio (INR) clinics if a patient’s warfarin dosing is unstable, in which case an alternative anticoagulant may be more appropriate.

### 14.2. Moderate to Severe COVID-19 Infection

In patients with a COVID-19 syndrome severe enough to be admitted to hospital, management of thrombotic risk requires a more proactive approach. Close monitoring is recommended for signs of either developing a VTE or coagulation abnormality and anticoagulation is recommended in the first instance, with or without mechanical prophylaxis.

Specific agents and dosing will differ, and these should be sought from local or national guidelines related to the place of treatment, and at the discretion of the clinician treating the patient. For instance, the National Institute of Clinical Excellence in the UK currently recommends that prophylactic low molecular weight heparin be offered to all COVID-positive patients without contraindications, unless already receiving a suitable prophylaxis agent, with several considerations for escalating to a short-term therapeutic dosing regimen [80]; however, the optimal dosing in patients without evidence of current VTE remains unclear [75].

Monitoring of biochemical markers is recommended for inpatients, not only for disease progression, but for signs of coagulopathy. Raised D-dimer levels are associated with increased morbidity and mortality [72,78] and should be monitored at 2–3 day intervals [75]. Though it is not generally recommended to dose anticoagulation treatment based on D-dimers [80], this can be used to identify a deteriorating patient, or the presence of thrombus development.

Severe COVID-19, as with many other critical conditions, represents an increased risk of developing DIC [72]. Close clinical and biochemical monitoring of signs of DIC is recommended in the form of platelet counts and coagulation panels, including fibrinogen levels and D-dimers. Decisions on the anticoagulation should be on a patient-to-patient basis, with considerations balanced between the risks of thrombosis and bleeding. It is generally recommended that prophylactic anticoagulation be continued in the absence of overt bleeding [75], and most reports suggest that incidence of life-threatening bleeding is less than that of major thrombosis [72]. Consultation with a local haematologist is advised to optimise care in such cases.

## 15. Management Recommendations in Dengue

### General Aspects of Management of Dengue

There are three phases of clinical presentations of dengue infections: febrile, critical (leakage) and convalescence (Figure 1). The convalescent phase can be divided into early (24–36 h after shock or 48–60 h after leakage) and a later convalescence phase (36 h after shock or 60 h after leakage). Disease manifestations are DHF (grades I and II) and DSS (grades III and IV). Expanded dengue syndrome is considered the most severe because this can lead to complications and death if not managed in a timely manner. Patients with DHF and DSS have different clinical presentations from other patients infected with dengue. Plasma leaks occur during the critical phases of DHF and DSS [81]. Most patients with dengue present with an undifferentiated febrile illness, but DHF/DSS occurs in only a small number of patients. Early diagnosis of DHF/DSS is important to initiate management promptly to prevent shock, severe illness and death. Symptomatic and supportive treatment is only needed during the early febrile phase. Dengue patients usually have high persistent fevers for 4–5 (range 2–7) days. Common signs and symptoms are severe headache, retro-orbital pain, myalgia, arthralgia and minor haemorrhagic manifestations such as petechial rash, epistaxis, gum bleeding and coffee-ground vomiting. Haematemesis and melena are both common symptoms. Haemoglobinuria is common on urine testing of haemolytic anaemias, especially in cases of thalassemia, other haemoglobinopathy and G-6-PD deficiency. Erythematous or maculopapular or petechial rash is especially common in adults. Nausea and vomiting along with poor appetite and malaise are common but nonspecific symptoms [82].

The use of an early tourniquet when diagnosing DHF and DSS is very helpful, where blood pressure is measured using an appropriately sized cuff. Cuff pressure is increased to a value that is mid-way between systolic and diastolic pressure for 5 min, and the cuff is then released. Results can be obtained after 1 min, or once normal skin circulation is noted. The test is regarded as positive if there are ≥310 petechiae/mm^3^ [83]. Paracetamol is the only recommended antipyretic to treat fever, both in children and in adults; non-steroidal anti-inflammatory drugs and aspirin are contraindicated. Anti-emetics are allowed for patients with nausea and vomiting. Other supportive and symptomatic medicines may be provided at the physician’s discretion, depending on clinical signs and symptoms. Some examples include anticonvulsants, anti-histamines and gastro protectives such as proton pump inhibitors. Antibiotics are not indicated unless superimposed secondary bacterial infections are suspected.

Shock or impending shock should be suspected in dengue patients with narrow pulse pressure (≤20 mmHg), hypotension, those with clinical signs of shock (rapid and weak pulse, mottled, cold and calmy skin, delayed capillary refill time (>3 s)), thrombocytopenia and raised haematocrit (320%), leukopenia, which can be related to a poor appetite, clinical deterioration or significant bleeding during defervescence. Any patient with shock or impending shock needs urgent hospitalisation for intensive monitoring of vital signs and prompt management [84].

## 16. Management of Haemorrhage in Dengue

The risk of clinically significant bleeding in dengue is unpredictable and often contributes to adverse outcomes. A large systematic review of 11 studies on prophylactic and therapeutic interventions for bleeding in dengue failed to identify any effective intervention in preventing or treating clinically significant bleeding in dengue [85].

A retrospective study evaluated 256 patients with dengue infection who developed thrombocytopenia (platelet count, <20 × 10^3^ platelets/µL) without prior bleeding, of which 188 received platelet transfusions. Subsequent bleeding, platelet increases, and platelet recovery times were similar between patients either receiving or not receiving platelet transfusions. Prophylactic platelet transfusion did not prevent bleeding in adult patients with dengue infection [86]. Another multicentre, open-label, randomised controlled trial (RCT) assigned 372 patients to transfusion (*n* = 188) or control (*n* = 184) groups. The intention-to-treat analysis shows clinical bleeding by day 7 or at hospital discharge occurred in 40 (21%) patients in the transfusion group and 48 (26%) patients in the control group (risk difference −4.98% (95% confidence interval (CI) −15.08 to 5.34); relative risk 0.81 (95% CI 0.56 to 1.17); *p* = 0.16); however, significantly more adverse events occurred in the transfusion group (5.81% (95% CI –4.42 to 16.01) versus 6.26% (95% CI 1.43 to 27.34); *p* < 0.01). No deaths were reported. The authors concluded that prophylactic platelet transfusion was not superior to supportive care in preventing bleeding in adult patients with dengue and thrombocytopenia, and could be associated with adverse events [87].

Some treatment recommendations are outlined below [85]:

1. Prophylactic platelet transfusion should not be routinely prescribed based on low platelet counts in patients with dengue and no bleeding.
2. Therapeutic platelet transfusion should not be routinely prescribed in patients with dengue with thrombocytopaenia and mild bleeding.
3. There is insufficient evidence to support or refute the use of platelet transfusion in patients with severe bleeding in dengue.
4. There is a need for further, well-designed RCTs to evaluate the role of platelets and plasma transfusion in patients in both the prevention of bleeding and in the setting of clinically significant bleeding in dengue infection.
5. There is currently insufficient evidence regarding the role of rFVIIa, anti-D globulin, Ig or tranexamic acid in the prevention or treatment of bleeding in dengue infection and there is a need for further research on these therapeutic agents.

## 17. Conclusions

Significant coagulation abnormalities, including thrombocytopenia, are common in both dengue and COVID-19 infections. This review discusses their common pathogenesis, clinical features including potential serological false positivity, and diagnostic challenges especially in dengue-endemic areas. We also review the importance of not transfusing platelets routinely as this can further stress already stretched services globally.

There are multiple complex mechanisms responsible for coagulation disturbances, thrombocytopenia and platelet dysfunction in dengue and paradoxical thromboembolism in COVID-19. The important aspects of treating COVID-19-induced immunothrombosis associated with thrombocytopenia are anticoagulation with or without aspirin. However, aspirin, nonsteroidal anti-inflammatory agents and anticoagulants are contraindicated in dengue infections.

The importance of recognising the similar clinical presentations of both diseases and excluding COVID-19 in the differential diagnoses in the setting of a dengue pandemic is paramount in preventing potential serious consequences in the current management of DENV-induced thrombocytopenia. While both dengue fever and COVID-19 infections can result in thrombocytopenia and coagulopathy, their clinical manifestations and management are quite different. Prevention and control strategies combined with vaccinations are key to controlling disease burden. While successful vaccinations for dengue currently remain largely ineffective, COVID 19 vaccinations have largely been successful.

## Figures and Tables

**Table 1:** Comparing dengue and COVID-19, based on data from Henrina et al. [7] and Centers for Disease Control and Prevention guidance [8].

General Features:	Dengue	COVID-19
**Virology**		
Family	Flaviviridae	Coronaviridae
Diameter	50 nm	65–125 nm
Genetic Material	ssRNA	ssRNA
**Presentation**		
Incubation	3–10 days	2 to 14 (median 4–5) days
Fever	Saddleback fever (with 2 peaks)	No specific fever patterns. Defervescence after 6 days of illness
Headache	45–95%	6.5–13.6%
Myalgia	12%	15–44%
Cough	21.5%	76%
Dyspnoea	9.5–95.2%	55%
Diarrhoea	6%	2–34%
Abdominal pain	17–25%	2%
Vomiting	30–58%	4–5%
Cutaneous manifestation	Skin flushing that blanch on pressure, petechiae, and convalescent rash	Erythematous rash, urticaria, chickenpox-like vesicles
Warning signs:	Persistent vomiting, mucosal bleeding, difficulty in breathing, lethargy/restlessness, postural hypotension, liver enlargement and progressive increases in haematocrit	Difficulty in breathing, persistent pain or pressure in the chest, new confusion, inability to wake or stay awake, bluish lips or face
**Laboratory Findings**		
Thrombocytopenia	69.51–100%	12–36.2%
Leukopenia	20–82.2%	25–29%
Lymphopenia	63%	63%
Raised AST	63–97%	31–35%
Raised ALT	45–97%	24–28%
Raised D-dimer	13–87%	46.4%

**Table 2:** Risk factors for severe dengue and COVID-19, based on Centers for Disease Control and Prevention guidance [27,28,29].

	Dengue	COVID-19
**Viral characteristics**	Viral titer correlates with disease severity. There may be strain and serotype differences in pathogenicity.	Relationship between viral titer and severity poorly understood. Certain variants, (via increased transmission, vaccine resistance etc.).
**Host factors**	Age (infant) Women, especially pregnant women. Patients with chronic medical conditions, including diabetes, asthma, obesity and heart disease. Patients with secondary DENV infection.	Age (elderly) Pregnant/recently pregnant women. Comorbidities, such as chronic kidney disease, malignancy, chronic lung disease, dementia, cardiovascular disease, diabetes, immunosuppression, multiple comorbidities.

**Table 3:** Coagulation disorders in COVID-19 and dengue (based on Iba et al. [67]).

General	Dengue	COVID-19
**Basic comparisons**	Consumptive coagulopathy is common	Consumptive coagulation disorder is seen in limited cases
**Bleeding in VHF and thrombosis in COVD-19**	Increased permeability in viral haemorrhagic fever also induces coagulation defects that can result in critical bleeding. The systemic viral infection also induces an acute inflammatory and hypercoagulable state causing DIC	COVID-19 is characterised by a high prevalence of thrombotic complications. Infrequently, bleeding can occur, especially in advanced stages of critical illness
**Pathogenesis**	Infected dendritic cells and macrophages lose their ability to regulate type I IFN levels, and lymphocytes undergo cell death. Inappropriate dendritic cell function perturbs the innate immune system and increases vascular permeability. Furthermore, the replicated viruses disseminate throughout the body to systemic reactions such as dysfunction of the visceral parenchymal cells, platelet disability and coagulopathy which lead to DIC resulting in uncontrolled haemorrhage	COVID-19 directly infects macrophages/monocytes, provoking inflammation and thrombosis by releasing proinflammatory cytokines, and expressing TF. Activated neutrophils eject neutrophil extracellular traps and disrupt antithrombogenicity by damaging glycocalyx. Thrombin activates endothelial cells, elicits a proinflammatory reaction, prothrombotic change and activates platelet aggregation. COVID-19 also infects endothelial cells by binding to ACE2 and stimulates the release of factor VIII, VWF and angiopoietin 2, resulting in thrombosis

## Data Availability

No new data was created or analysed in this study. Data sharing is not applicable to this article.
